# Amelioration of radiation-induced skin injury by adenovirus-mediated heme oxygenase-1 (HO-1) overexpression in rats

**DOI:** 10.1186/1748-717X-7-4

**Published:** 2012-01-17

**Authors:** Shuyu Zhang, Chuanjun Song, Jundong Zhou, Ling Xie, Xingjun Meng, Pengfei Liu, Jianping Cao, Xueguang Zhang, Wei-Qun Ding, Jinchang Wu

**Affiliations:** 1 School of Radiation Medicine and Protection, Medical College of Soochow University, Suzhou 215123, China; 2 Cultivation base of State Key Laboratory of Stem Cell and Biomaterials built together by Ministry of Science and Technology and Jiangsu Province, Soochow University, Suzhou 215006, China; 3 The Core Laboratory of Suzhou Cancer Center and Department of Radiotherapy of Suzhou Hospital Affiliated to Nanjing Medical University, Suzhou 215001, China; 4 Department of Gastroenterology, the Affiliated Jiangyin Hospital of Southeast University, Jiangyin 214400, China; 5 Department of Pathology, University of Oklahoma Health Sciences Center, Oklahoma City, 73104, USA

**Keywords:** Radiation-induced skin injury, HO-1, adenovirus, lipid peroxidation, apoptosis

## Abstract

**Objective:**

Radiation-induced skin injury remains a serious concern for radiation therapy. Heme oxygenase-1 (HO-1), the rate-limiting enzyme in heme catabolism, has been reported to have potential antioxidant and anti-apoptotic properties. However, the role of HO-1 in radiation-induced skin damage remains unclear. This study aims to elucidate the effects of HO-1 on radiation-induced skin injury in rats.

**Methods:**

A control adenovirus (Ad-EGFP) and a recombinant adenovirus (Ad-HO1-EGFP) were constructed. Rats were irradiated to the buttock skin with a single dose of 45 Gy followed by a subcutaneous injection of PBS, 5 × 10^9 ^genomic copies of Ad-EGFP or Ad-HO1-EGFP (n = 8). After treatment, the skin MDA concentration, SOD activity and apoptosis were measured. The expression of antioxidant and pro-apoptotic genes was determined by RT-PCR and real-time PCR. Skin reactions were measured at regular intervals using the semi-quantitative skin injury score.

**Results:**

Subcutaneous injection of Ad-HO1-EGFP infected both epidermal and dermal cells and could spread to the surrounding regions. Radiation exposure upregulated the transcription of the antioxidant enzyme genes, including *SOD-1*, *GPx2 *and endogenous *HO-1*. HO-1 overexpression decreased lipid peroxidation and inhibited the induction of ROS scavenging proteins. Moreover, HO-1 exerted an anti-apoptotic effect by suppressing *FAS *and *FASL *expression. Subcutaneous injection of Ad-HO1-EGFP demonstrated significant improvement in radiation-induced skin injury.

**Conclusions:**

The present study provides evidences for the protective role of HO-1 in alleviating radiation-induced skin damage in rats, which is helpful for the development of therapy for radiation-induced skin injury.

## Introduction

Radiotherapy offers valuable alternatives to primary surgical approaches for cancer patients. Despite being a useful modality for cancer therapy, ionizing radiation may injure surrounding normal tissues [1,2]. Although the skin is not the primary target, it may be significantly injured and its function profoundly impaired during radiation therapy [3,4]. While increased efforts have led to new treatment schedules that are designed to maximize antineoplastic effects and minimize skin toxicity, radiation-induced skin injury remains a serious concern, which may limit the duration of radiation and the dose delivered. In addition, nuclear accidents are another cause of such skin reactions [5,6]. Thus, the management of radiation-induced skin damage is critical for effective radiation therapy.

During radiation exposure, skin tissue damage occurs instantaneously, mediated by a burst of free radicals. Irradiated cells produce reactive oxygen species (ROS), including oxygen ions, free radicals, and peroxides. The detrimental ROS can further result in damages to nuclear DNA and alterations of proteins, lipids, and carbohydrates [7]. In response to ionizing radiation exposure, signal transduction pathways, transcription factors, DNA repair enzymes and antioxidant enzymes are activated. Many of these signaling and gene expression pathways are involved in intracellular metabolic redox reactions to buffer the ROS [8]. Meanwhile, inflammatory cells are recruited and fibrogenesis and angiogenesis are initiated. High-dose ionizing radiation finally culminates in cutaneous cell death and profound impairment of skin function [4].

Heme oxygenases (HO) are microsomal enzymes that catalyse the heme ring into carbon monoxide (CO), free iron and biliverdin. Biliverdin is rapidly converted to bilirubin by biliverdin reductase. CO and bilirubin have been well described as having antioxidant and anti-inflammatory properties [9,10]. The HO family consists of three homologous isoenzymes, an inducible HO-1, a constitutive HO-2 and an HO-3 with low enzymatic activity [11]. HO-1 is also known as heat shock protein 32 (HSP32), which is strongly induced by various stimuli including heat shock, metals, cytokines and oxidative stress [12,13]. In the skin, the expression of HO-1 is strongly induced by ultraviolet radiation by the generation of biologically active phospholipid oxidation products. And its expression is most pronounced in the epidermal layers immediately below the stratum corneum [14]. HO-1 is also involved in skin immunity. Cutaneous inflammation was enhanced by HO-1 inhibition and was abrogated by treatment with the HO-1 inducer cobaltic protoporphyrin (CoPP) [15].

Since radiation exposure produces oxidative stress and consequently leads to skin injury, we reasoned that HO-1 overexpression would ameliorate the radiation-induced skin damage. In this study, we constructed a control adenovirus (Ad-EGFP) and a recombinant adenovirus (Ad-HO1-EGFP) that can overexpress rat HO-1. Subcutaneous injection of this Ad-HO1-EGFP infected cells in the epidermis and dermis, and ameliorated radiation-induced skin injury by reducing oxidative stress and suppressing apoptosis. These results will help the development of curative measures for radiation-induced skin injury.

## Materials and methods

### Construction of recombinant adenovirus

The rat HO-1 (GenBank accession no. NM012580) coding region was amplified by PCR using a pair of primers: (forward) 5'- ATGGAGCGCCCACAGCT-3' and (reverse) 5'- TTACATGGCATAAATTCCCACTG -3' and rat HO-1 cDNA. The coding region of the rat *HO-1 *gene was initially cloned into the pIRES2-EGFP vector, and the sequence of HO-1-IRES-EGFP was then subcloned into pShuttle-CMV. Adenoviruses expressing HO-1-IRES-EGFP and the control EGFP were generated using AdEasy™ Adenoviral Vector System (Agilent Technologies, Santa Clara, CA) per the manufacture's protocols. In brief, Shuttle plasmid DNA was linearized using PmeI, and treated with alkaline dephosphatase. The fragment was purified and recombined with pAdEasy-1 in BJ5183 cells. Positive clones were selected and transformed into XL10-Gold supercompetent E. coli cells. The confirmed recombinant adenovirus plasmids were each digested with PacI. Plasmids were then transfected into HEK-293A cells. Adenovirus expressing HO-1-IRES-EGFP or EGFP was propagated in HEK-293A cells and purified by CsCl gradients by ultracentrifugation. Titer of the viral solution was determined by Adeno-X Rapid Titer kit (Clontech, Mountain View, CA). These viruses were stored at -80°C prior to injection. Rats were given subcutaneous injection of Ad-EGFP or Ad-HO1-EGFP at an amount of 5 × 10^9 ^PFU suspended in 200 μl phosphate-buffered saline (PBS) after radiation exposure.

### Animals and treatments

Male SD rats (4 weeks old) were purchased from Shanghai SLAC Laboratory Animal Co., Ltd. (Shanghai, China). Rats were anesthetized with an intraperitoneal injection of ketamine (75 mg/kg) and xylazine (10 mg/kg), and the hair on rat buttock skin was shaved using a razor. Rats were immobilized with adhesive tape on a plastic plate to minimize motion during irradiation exposure. A 3 cm thick pieces of lead was used to shield the rats and localize the radiation field (3 cm×4 cm). A single dose of 45 Gy was administered to the treatment area of each rat at a dose rate of 750 cGy/min using a 6-MeV electron beam accelerator (Clinac 2100EX; Varian Medical Systems Inc, CA). This dose was selected because it can significantly induce skin injury. After irradiation, rats were randomly divided into three groups: 1) rats were administered with a subcutaneous injection of 200-μl volume PBS; 2) with a subcutaneous injection of 5 × 10^9 ^genomic copies of Ad-EGFP in a 200-μl volume; 3) with a subcutaneous injection of 5 × 10^9 ^genomic copies of Ad-HO1-EGFP in a 200-μl volume. Skin reactions were followed at regular intervals using the semi-quantitative skin injury scale from 1 (no damage) to 5 (severe damage), as previously described [16]. All the protocols and procedures were approved by the Animal Experimentation Ethics Committee of the Soochow University.

### Fluorescent Imaging of Ad-HO1-EGFP *in vivo*

Before injection, rats were anesthetized with an intraperitoneal injection of ketamine (75 mg/kg) and xylazine (10 mg/kg). A viral load of 5 × 10^9 ^genomic copies of Ad-HO1-EGFP (200 μl) or 200 μl of PBS was subcutaneously injected into buttocks of SD rats, respectively. To visualize infected cells, three days after the injection, the rats were anesthetized and imaged using a Kodak In-Vivo Multispectral Imaging System FX. Then, rats were sacrificed, frozen-cut sections of buttock skin were visualized under a fluorescent microscope.

### Immunohistochemistry

Skin tissues were fixed in 10% neutral buffered formalin and later embedded in paraffin. Three micrometers thick paraffin sections were deparaffinized and heat treated with citrate buffer, pH 6.0, for 7 min following an epitope retrieval protocol. Endogenous peroxidase was blocked with 3% hydrogen peroxide for 15 min at room temperature, and tissue non-specific-binding sites were blocked with skimmed milk powder at 4% applied for 30 min. Sections were then incubated with the HO-1 antibody (Santa Cruz Biotechnology) for 1 h (dilution 1:200) and mixed with skimmed milk powder at 2% again to reduce unspecific staining. Biotinylated secondary antibody was then added for 30 min. Avidin-biotin-peroxidase complex (Dako LSAB2 system, DAKO Co., Carpinteria, CA) was added and color was developed using 3-3'-diaminobenzidine. Counterstaining was done with hematoxylin. All steps were performed at room temperature. Omitting the primary antibody from the procedure on the protocol was used as a negative control.

### RNA extraction, reverse transcription- (RT)-PCR and real-time PCR

Total RNA from skin tissues was extracted with Trizol (Invitrogen, Carlsbad, CA) and reversely transcribed into cDNA using an oligo(dT)_12 _primer and the Superscript II reverse transcriptase (Invitrogen). SYBR green dye (Takara Bio Inc., Shiga, Japan) was used for amplification of cDNA. mRNA levels of *SOD-1*, endogenous *HO-1*, *Catalase, GPx2, FAS, FASL *and the internal standard *β-Actin *were measured by RT-PCR and real-time quantitative PCR in triplicate using a Prism 7500 real-time PCR machine (Applied Biosystems, Foster City, CA). The primer sequences were listed in Table 1.

**Table 1:** Primer sequences for RT-PCR and real-time PCR analysis

Gene	Forward primer	Reverse primer	Product size (bp)
*β-Actin*	5'- CCCATCTATGAGGGTTACGC -3'	5'- TTTAATGTCACGCACGATTTC -3'	150
*endogenous HO-1*	5'-CAGAAGGGTCAGGTGTCCAG-3'	5'- GAAGGCCATGTCCTGCTCTA -3'	262
*SOD-1*	5'- GCCAATGTGTCCATTGAAGA -3'	5'- CAATCACACCACAAGCCAAG -3'	168
*CAT*	5' - CACTGACGTCCACCCTGAC -3'	5'- GACTCCATCCAGCGATGATT -3'	242
*GPx2*	5' - CCTCGCTCTGAGGAACAACT-3'	5'- TGCCCATTGACATCACACTT -3'	220
*FAS*	5'- AGCTGCTCCAGTGCTGGTAT -3'	5'- CATAGGTGGCAGGCTCTCTC -3'	232
*FASL*	5'-AGACCACAAGGTCCAACAGG-3'	5'- CAAGTAGACCCACCCTGGAA-3'	244

### Malondialdehyde (MDA) concentration measurement

After excision, fresh skin samples were homogenized with 50 mM phosphate buffer (pH 7.4). Then, homogenates were centrifuged at 12,000 × g for 10 min at 4°C. Supernatants were separated and kept at -20°C until MDA measurements were performed. Protein concentration supernatants were determined according to Lowry assay [17] using bovine serum albumin as a standard.

Tissue MDA levels were determined by thiobarbituric acid (TBA) reaction. The optical density (OD) was measured at a wavelength of 532 nm. The assay depended on the measurement of the pink color produced by the interaction of barbituric acid with MDA generated as a result of lipid peroxidation. The colored reaction with 1,1,3,3- tetraethoxy propane was used as the primary standard. The MDA levels were determined by the method of Yagi *et al. *[18]. MDA levels were expressed as a nanomol per milligram of protein (nmol/mg protein).

### SOD activity measurement

Activity of SOD was measured using commercial available kit purchased from Nanjing Jiancheng BioEngineering (Nanjing, China). SOD activity measurement was based on the instructions of the colorimetric method.

### Terminal deoxynucleotidyl transferase dUTP nick-end labeling (TUNEL) assay

Five μm skin sections were deparaffinized in xylene and hydrated in decreasing concentrations of ethanol, and terminal deoxynucleotidyl transferase dUTP nick-end labeling (TUNEL) assay was performed following manufacturer's instructions (Keygen, Nanjing, China). Ten random fields from 4 slides per group were examined. The TUNEL-positive brown nuclei within the skin were counted. The data were expressed as the mean number of apoptotic cells/high power field.

### Statistical analysis

Data were expressed as the mean ± standard error of the mean (SEM) of at least three independent experiments. Standard error bars are included for all data points. The data were first analyzed with the Kolmogorov-Smirnov test for data distribution normality and then analyzed using Student's t test when only two groups were present, or assessed by one-way analysis of variance (ANOVA) when more than two groups were compared. For ordinal data, the Mann-Whitney test was used. Statistical analysis was performed using SPSS software (Release 17.0, SPSS Inc.). Data were considered significant if *P *< 0.05.

## Results

### The infection and expression of Ad-HO1-EGFP after its subcutaneous administration

**Figure 1:**
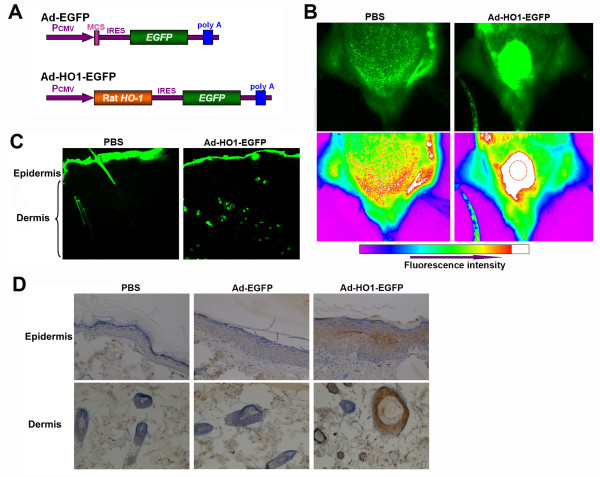
**The distribution and expression of Ad-HO1-EGFP in rat skin**. (A) Schematic diagram of the recombinant Ad-HO1-EGFP vector and the control vector (Ad-EGFP). (B) *In vivo *imaging of EGFP expression in SD rats. Rats were injected subcutaneously with 5 × 10^9 ^genomic copies of Ad-HO1-EGFP or equivalent volume of PBS. Three days after injection, the expression of EGFP was visualized. The dotted circle indicates the injection region. (C) The expression of EGFP was imaged from frozen-cut sections and observed under a fluorescent microscope (× 40). (D) Immunohistochemistry analysis of HO-1 expression in rat epidermis and dermis (× 200).

To illustrate the therapeutic role of HO-1 in radiation-induced skin injury, a recombinant adenovirus (Ad-HO1-EGFP) and a control adenovirus (Ad-EGFP) were constructed (Figure 1A). To examine adenovirus mediated *HO-1 *gene expression and distribution *in vivo*, Ad-HO1-EGFP (5 × 10^9 ^genomic copies) was subcutaneously injected into buttocks of SD rats. After 3 days, *EGFP *gene expression was observed using a Kodark *in vivo *imaging system. Highly intensive EGFP expression was observed in the Ad-HO1-EGFP-injected buttock of SD rat, compared to the PBS-injected buttock. Interestingly, as shown in Figure 1B, fluorescence could be observed in the surrounding skin regions of the injection site (dotted circle), suggesting that Ad-HO1-EGFP could spread and infect surrounding skin cells. To further investigate the distribution of EGFP expression in skin tissues, frozen-cut sections of the buttock skin were observed under a microscope. As shown in Figure 1C, EGFP expression was observed in dermis of Ad-HO1-EGFP-injected rats, but not in that of the PBS-injected rats. Nevertheless, it is unclear whether Ad-HO1-EGFP infected the epidermis of rats because the stratum corneum displayed strong fluorescence, even in the control group. EGFP expression was found to persist for at least two weeks (data not shown). Immunohistochemistry was used to confirm the expression of HO-1 in rat skin tissues. As shown in Figure 1D, the expression of HO-1 was more pronounced in dermis (hair follicles and sebaceous glands) than in epidermis, 3 days after Ad-HO1-EGFP administration.

### HO-1 overexpression decreased the production of MDA and the induction of antioxidant enzymes

**Figure 2:**
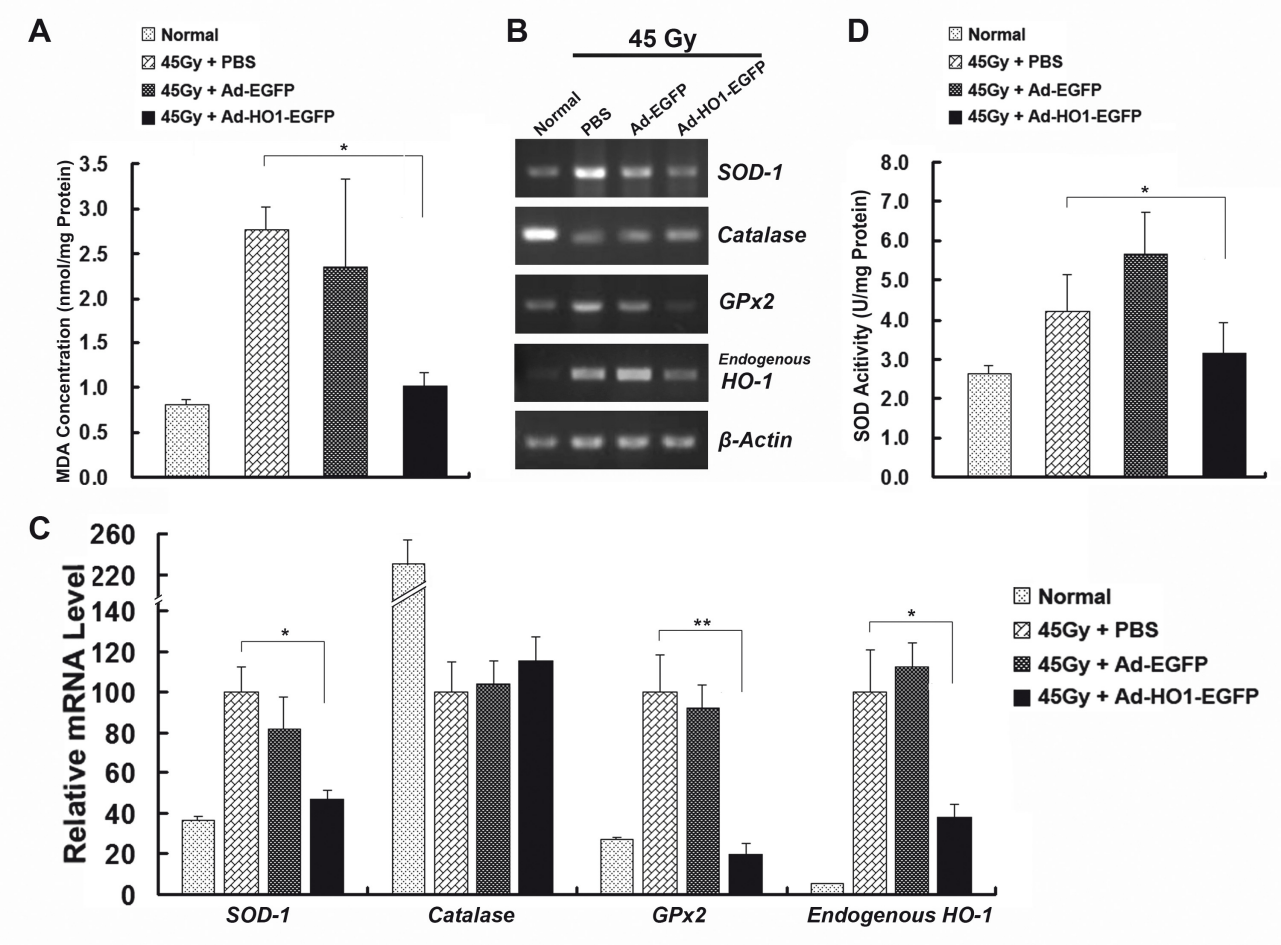
**Quantitative analyses of MDA concentration, SOD activity and relative mRNA levels of the antioxidant enzyme genes**. (A) Relative MDA concentration in rat skin tissues of indicated groups. (B) SOD activity in rat skin tissues of indicated groups. (C) Representative reverse transcription (RT)-PCR analysis of the antioxidant enzyme genes in indicated groups. (D) Real-time PCR analysis of the antioxidant enzyme genes in indicated groups (4 animals per group). mRNA levels are presented as means ± SEM (normalized to *β-Actin*). The men expression level in PBS-injected control group was arbitrarily set as 100%. * *P *< 0.05; ** *P *< 0.01, compared with PBS-injected control group.

Since radiation-induced ROS results in oxidative damage in lipids, DNA and proteins, cellular defenses have been proposed to play important roles in protecting the skin cells against oxidative stress and reducing the progression of skin injury [19,20]. To test whether overexpression of HO-1 affects the radiation-induced lipid peroxidation, the concentration of MDA in the skin tissues after radiation with 45Gy was measured. As shown in Figure 2A, MDA levels were significantly decreased in tissues infected with Ad-HO1-EGFP, compared with the control group (*P *< 0.05), whereas infection of Ad-EGFP did not reduce the production of MDA. This indicated that HO-1 overexpression attenuates radiation-induced lipid peroxidation.

A series of enzymes is involved in counteracting oxidative stress in cells, including superoxide dismutases (SOD), Catalase and Glutathione peroxidase (GPx). We found that 45Gy irradiation strongly upregulated mRNA expression of *SOD-1*, *GPx2 *and endogenous *HO-1*, and that overexpression of HO-1 significantly attenuated expression of these enzymes, compared with PBS-injected group (Figure 2B and 2C). Interestingly, radiation inactivated the expression of Catalase, relative to control skin tissues. The effect of HO-1 overexpression on SOD activity was also measured. Consistent with SOD-1 mRNA level, radiation increased the SOD activity, and Ad-HO1-EGFP injection counteracted this increase (Figure 2D). These results suggested that HO-1 overexpression significantly attenuates radiation-induced intracellular ROS accumulation, thereby reducing the need for skin cells to elicit an oxidant-induced response.

### HO-1 overexpression inhibited the apoptosis of skin cells

**Figure 3:**
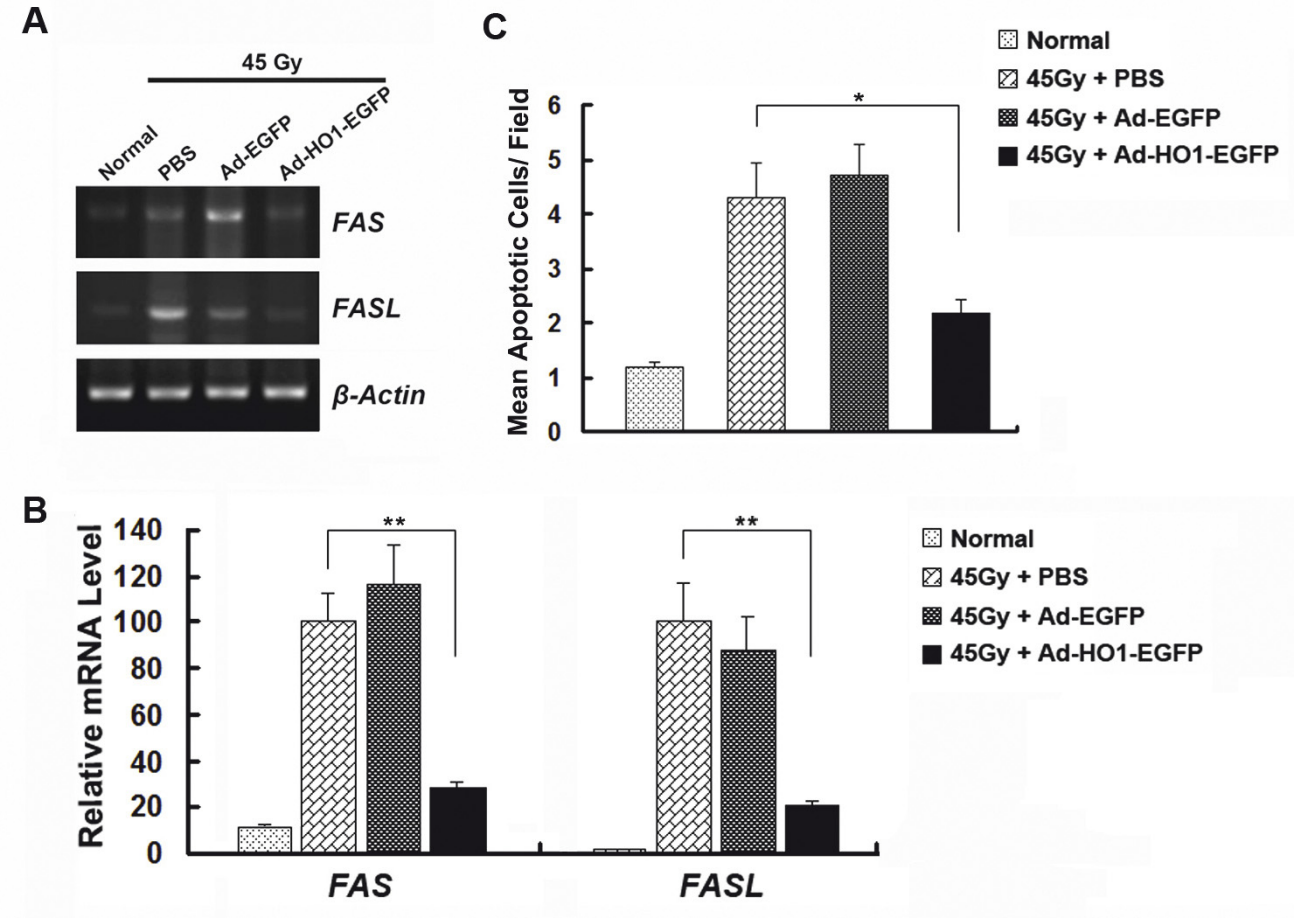
**Effects of HO-1 overexpression on radiation-induced skin cell apoptosis**. (A) Representative RT-PCR analysis of the apoptosis pathway genes in rat skin tissues of indicated groups. (B) Real-time PCR analysis of the apoptosis pathway genes in indicated groups (4 animals per group). mRNA levels are presented as means ± SEM (normalized to *β-Actin*). The men expression level in PBS-injected control group was arbitrarily set as 100%. (C) Quantification of mean TUNEL-positive cells/per field are expressed as mean ± SD. * *P *< 0.05; ** *P *< 0.01, compared with PBS-injected control group.

To investigate the effect of HO-1 overexpression on cell apoptosis in the rat skin, we measured the mRNA levels of the key apoptosis-related genes. The cell death receptor Fas/CD95 and Fas/CD95 ligand (Fas-L) are complementary receptor-ligand proteins that initiate extrinsic apoptotic pathway [21]. After 45 Gy radiation, mRNA levels of *FAS *and *FASL *showed a marked elevation in skin tissues, suggesting an initiation of the apoptosis cascade. Administration of Ad-HO1-EGFP inhibited the expression of *Fas *and *FASL*, compared with the PBS- or Ad-EGFP-injected group (Figure 3A and 3B). Consistently, results from TUNEL assay showed significantly lower apoptotic cell death in Ad-HO1-EGFP treated group, compared with the PBS-treated rats (Figure 3C). These results indicated that HO-1 overexpression reduces radiation-induced apoptosis in skin cells.

### HO-1-overexpression ameliorated radiation-induced skin injury

**Figure 4:**
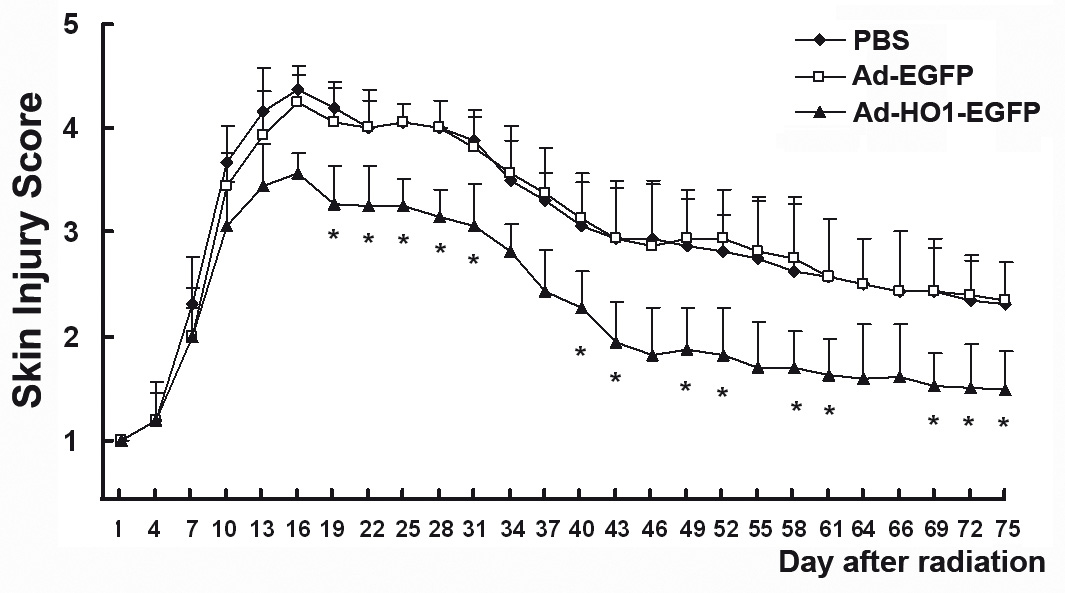
**Effect of HO-1 overexpression on the amelioration of radiation-induced skin injury**. Rats were irradiated to the buttock skin with a single dose of 45 Gy followed by an injection of PBS, 5 × 10^9 ^genomic copies of Ad-EGFP or Ad-HO1-EGFP (8 animals per group). Skin injury in these groups was measured using a semi-quantitative score of 1 (no damage) to 5 (severe damage). * *P *< 0.05, compared with PBS-injected control group.

To determine whether adenovirus mediated HO-1 expression could attenuate radiation-induced skin injury, we performed an experiment using a single dose of 45 Gy delivered to the buttock skin of SD rats followed by a subcutaneous injection of Ad-EGFP or Ad-HO1-EGFP (5 × 10^9 ^genomic copies). The rats in the control group were injected with equivalent volume of PBS. Injuries of skin tissues were graded on a scale of 1 (no damage) to 5 (severe damage). Cutaneous damage was noticed 4 days after irradiation. Skin injury reached a maximum 15-18 days after irradiation, and then the skin wounds began to heal. Radiation-induced skin injury was significantly less severe in the Ad-HO1-EGFP-injected group, compared to the PBS-treated rats 19 days post irradiation, while the Ad-EGFP-administrated rats showed similar skin tissue damage with that of the control group (Figure 4).

## Discussions

There is increasing evidence indicating that HO-1 is involved in the pathogenesis of many diseases. HO-1 is a stress-responsive enzyme that can be induced by various oxidative agents and has recently emerged as a crucial mediator of antioxidant forces and tissue-protective actions. The physiological importance of HO-1 has been confirmed in HO-1 knockout-mice and in a HO-1-deficient human case, both of which showed a reduced cellular resistance to oxidative stress [22-24]. Due to this documented protective activity of HO-1, we constructed an adenovirus construct that was used to overexpress HO-1 in rats aiming at testing its role in radiation-induced skin damage. We found that subcutaneous injection of the recombinant adenovirus ameliorates radiation-induced skin injury in rats most likely via suppression of oxidative stress and inhibition of cutaneous cell apoptosis.

Radiation exposure to tissues generates ROS and oxidative stress, which will trigger inflammatory response and cell death in affected areas. The ROS is known to oxidize fatty acids generating highly toxic lipid peroxides that lead to apoptotic cell death [25]. There is a well-established antioxidant enzyme system in eukaryotic cells which protects cells from oxidative damages. For instance, SOD is a primary antioxidant enzyme that converts superoxide to hydrogen peroxide, which can be subsequently detoxified into water and oxygen by catalase and GPx [26]. SOD-1 is the predominant form of three SOD enzymes, and GPx2 is a member of the selenium-containing antioxidative enzyme family, acting directly as an antioxidant and an inhibitor of lipid peroxidation [27]. In the present study, we demonstrated that injection of Ad-HO1-EGFP reduced radiation-induced skin MDA levels indicating that HO-1 attenuates radiation-induced lipid peroxidation. Furthermore, we found that radiation-induced expression of *SOD-1*, *GPx2 *and endogenous *HO-1 *was significantly down-regulated in tissues of the rats that were injected with Ad-HO1-EGFP. This suggests that HO-1 inhibits antioxidative responses from the skin cells to radiation insults. Note that overexpression of SOD-1 has been described to likely induce cell apoptosis [28], which may be associated with accumulation of toxic hydrogen peroxide produced by SOD that cannot be further detoxified by catalase and GPx. In this context, reduced SOD-1 expression supports a protection action of HO-1 on radiation-induced skin injury. However, radiation induced a suppression of catalase expression in our model system, which is in contrast to the expression of other antioxidant enzymes, and HO-1 over-expression had no effects on this suppression. A previous study by Chang *et al. *also reported that UVB radiation inhibited catalase activity in skin cells that was gradually recovered [29]. The underlying mechanisms for radiation-induced inactivation of catalase in skin cells merit further investigation. Taken together, these results indicate that overexpression of HO-1 attenuates oxidative stress and inhibits progression of skin injury.

Skin apoptosis is considered to be a vital indicator for skin injury [30]. The mechanisms by which apoptosis occurs in radiation-induced skin injury may provide an insight into the development of future radiation therapy. FAS and FASL serve as external pro-apoptotic sensors of cell damage and transmit the signal to the internal effectors. Accumulating evidence suggests that radiation-induced skin injury is mediated by Fas and FasL [31-33]. We found that nonirradiated skin had little Fas and FasL expression; however, after electron beam exposure, the expression levels of Fas and FasL were strongly induced, which is in line with a previous report using ultraviolet radiation [31]. We demonstrated that radiation promoted apoptosis of rat cells in the epidermis and dermis using the TUNEL assay, and HO-1 overexpression resulted in a significant resistance to apoptosis, evidenced by a diminution of the TUNEL-positive cells. Thus, the alleviated damage in Ad-HO1-EGFP treated rats is likely due to less apoptosis of the skin cells. Suppression of apoptosis by HO-1 may contribute to alleviating the severity of skin injury induced by radiation.

In recent years, pharmacological interventions have been reported to reduce toxic radiation reactions. Pretreatment with caffeine, a major component of coffee, delays the progression of radiation-induced skin reactions, but cannot attenuate injury severity [34]. Essential fatty acids are shown to modulate normal tissue reactions to radiation damages [35]. It was reported that histone deacetylase (HDAC) inhibitors can suppress the aberrant expression of radiation-induced transforming growth factor β and tumor necrosis factor α and promote the healing of skin wounds [36]. In comparison, adenovirus-mediated gene transfer confers a long-term effect with high expression levels of the genes delivered and gene-specific activity. Since ROS are involved in radiation-induced injury, supplementations of antioxidant enzymes have been utilized to mitigate this injury. It is well known that detrimental superoxide anion, one of the major damaging ROS, can be converted to hydrogen peroxide by SOD. Yan *et al. *mitigated radiation-induced skin injury by AAV-mediated MnSOD (SOD2) expression using a mouse model [37]. MnSOD has also been shown to decrease superoxide levels and protect the bladder from radiation damage [38]. Our results indicate an alternative to SOD in the treatment of radiation-induced injury. Since HO-1 and SOD-1 exert their antioxidant actions via different cellular mechanisms, whether a combination of SOD and HO-1 delivery could be more effective in reducing radiation-induced skin damage warrants further exploration.

## Conclusions

In summary, subcutaneous treatment with Ad-HO1-EGFP attenuated radiation-induced skin injury in rats via suppression of lipid peroxidation and apoptotic cell death. The present study provides evidences for the first time showing the protective role of HO-1 in radiation-induced skin injury in rats. This report contributes to our understanding of the therapeutic potential of HO-1 for radiation-induced skin injury.

## Abbreviations

HO-1: heme oxygenase-1; EGFP: enhanced green fluorescent protein; RT-PCR: reverse transcription-polymerase chain reaction; MDA: malondialdehyde; SOD: superoxide dismutases; GPx: Glutathione peroxidase; MDA: malondialdehyde

## Conflict of interest Statement

The authors declare that they have no competing interests.

## Authors' contributions

SZ carried out the molecular biology studies and drafted the manuscript. CS and JZ performed the animal experiment. LX, XM and PL and performed the statistical analysis. JW, WD, JC and XZ participated in study design and coordination and helped to draft the manuscript. All authors read and approved the final manuscript.
